# PCSK9 participates in oxidized‐low density lipoprotein‐induced myocardial injury through mitochondrial oxidative stress and Drp1‐mediated mitochondrial fission

**DOI:** 10.1002/ctm2.729

**Published:** 2022-02-20

**Authors:** Xuan Li, Fangjie Dai, Hao Wang, Ge Wei, Qiu Jiang, Peipei Yin, Shijun Wang, Junbo Ge, Cheng Yang, Jian Wu, Yunzeng Zou

**Affiliations:** ^1^ Shanghai Institute of Cardiovascular Diseases Zhongshan Hospital and Institutes of Biomedical Sciences, Fudan University Shanghai China; ^2^ Department of Laboratory Medicine Zhongshan Hospital, Fudan University Shanghai China; ^3^ Department of Cardiac Surgery Shanghai Institute of Cardiovascular Diseases, Zhongshan Hospital, Fudan University Shanghai China; ^4^ Department of Cardiology Affiliated Hospital of Guizhou Medical University Guiyang China

Dear Editor,

Oxidized low‐density lipoprotein (ox‐LDL), derived from low‐density lipoprotein (LDL) under oxidative stress, is a crucial risk factor in the initiation and development of various cardiovascular diseases.^1^ Although we have quite recently reported that ox‐LDL/LOX‐1 (lectin‐like oxidized low‐density lipoprotein receptor‐1) induced cardiac hypertrophy in mice,^2^ whether ox‐LDL impairs cardiac function directly in vivo remains elusive. In this study, we reveal that proprotein convertase subtilisin/kexin type 9 (PCSK9) contributes to ox‐LDL‐LOX‐1‐induced myocardial damage via increasing oxidative stress and mitochondrial fission.

PCSK9, a liver‐derived secretory protease, promotes the LDL‐R degradation to maintain cholesterol homeostasis.^3^ Besides the modulating of LDL and ox‐LDL receptor turnover, PCSK9 is constitutively expressed in cardiomyocytes^4^ and is implicated to exert extrahepatic effects in the heart.^5–7^ In this study, C57BL/6J mice subjected to intraperitoneal injection of ox‐LDL for 4 weeks showed significant cardiac hypertrophy and dysfunction (Figure S1), prominent myocardial apoptosis (Figure S2A,B), increased myocardial oxidative stress and Drp‐1‐mediated mitochondrial fission (Figure S3A–D), accompanied by elevated PCSK9 levels in serum and heart (Figure 1B,D). Similarly, in cultured adult and neonatal mouse ventricular myocytes (AMVMs and NMVMs), ox‐LDL also induced oxidative stress (Figure S3E,G), mitochondrial dysfunction (Figure S3F,H,I–J), apoptosis (Figure S2C,D) and PCSK9 upregulation (Figure 1C,E; Figure S4A). Furthermore, a positive correlation between ox‐LDL and PCSK9 levels was obtained in the serum of patients with hyperlipidemia and heart failure (Figure 1A). To confirm the involvement of PCSK9 in cardiac hypertrophy and function, a PCSK9 inhibitor evolocumab was administered to ox‐LDL‐treated mice. No significant difference was observed in cardiac hypertrophy or function between control mice and evolocumab‐administrated mice. Administration of evolocumab alleviated the hypertrophic response in ox‐LDL‐treated hearts, as revealed by reduced LV internal dimension, LV wall thickness (Figure 1F), HW/BW (Figure 1H), cardiomyocyte size (Figure 1I), levels of Hypertrophy gene (Figure 1J) and fibrosis (Figure S4B). In addition, evolocumab improved cardiac function, as demonstrated by increased LVEF (Figure 1F), max dP/dt and min dP/dt (Figure 1G). These data indicate that cardiac hypertrophy and dysfunction induced by ox‐LDL were attenuated in evolocumab‐administered mice compared to control mice.

**FIGURE 1:**
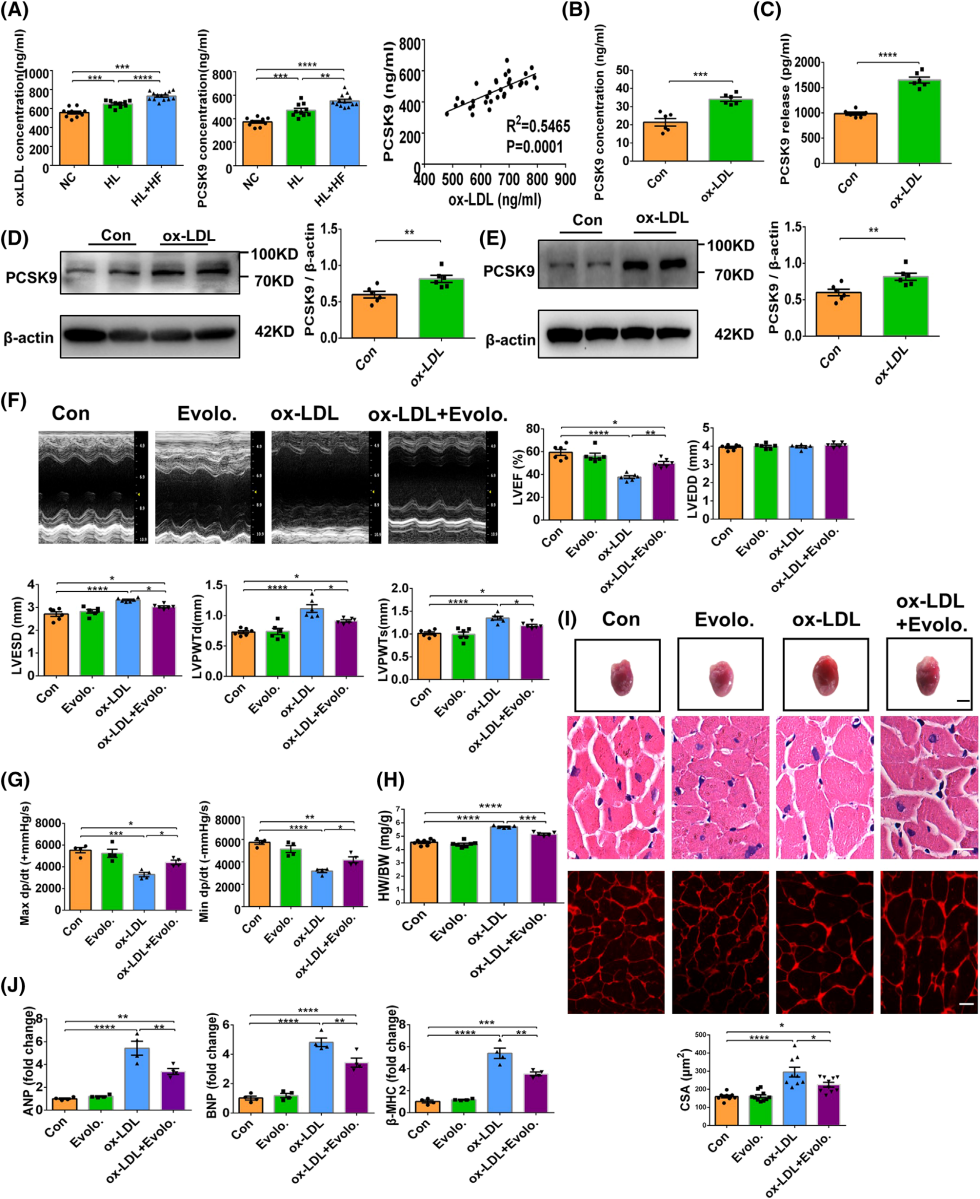
Oxidized low‐density lipoprotein (ox‐LDL) elevates the expression of PCSK9 and inhibition of PCSK9 alleviates ox‐LDL‐induced cardiac dysfunction. (A) Serum levels of ox‐LDL and PCSK9 in patients with hyperlipidaemia and heart failure (HL+ HF), patients with hyperlipidaemia (HL) and control subjects (NC) were measured by ELISA kit, *n* = 10–13/group (one‐way ANOVA followed by Tukey's multiple comparisons test). And correlation analysis of ox‐LDL and PCSK9 was shown. (B) Serum levels of PCSK9 in ox‐LDL‐treated mice by ELISA, *n* = 6 (Student's *t*‐test). (C) PCSK9 levels in the supernatants from NMVMs were determined by ELISA, *n* = 6 (Student's *t*‐test). (D) Western blot analysis of PCSK9 in ox‐LDL‐treated hearts. The quantification is shown, *n* = 6 (Student's *t*‐test). (E) Western blot analysis of PCSK9 in ox‐LDL‐treated NMVMs. The quantification is shown, *n* = 6 (Student's *t*‐test). (F) Representative M‐mode echocardiography. And LVEF, LVEDD, LVESD, LVPWTd and LVPWTs were analysed by echocardiography, *n* = 6 (one‐way ANOVA followed by Tukey's multiple comparisons test). (G) Maximal +dp/dt and minimal –dp/dt. *n* = 4 (one‐way ANOVA followed by Tukey's multiple comparisons test). (H) Heart weight and body weight ratio (HW/BW), *n* = 6 (one‐way ANOVA followed by Tukey's multiple comparisons test). (I) Cross‐sectional area (CSA) of cardiomyocytes. *n* = 25‐30/mouse, 3 mice/group (one‐way ANOVA followed by Tukey's multiple comparisons test). Gross morphology and histological analysis of heart tissues. Upper panel: gross morphology, scale bars, 6.0 mm; middle panel: HE staining, scale bars, 20 μm; lower panel: Wheat germ agglutinin (WGA) staining, scale bars, 10 μm. *n* = 6 (one‐way ANOVA followed by Tukey's multiple comparisons test). (J) *ANP*, *BNP* and *β‐MHC* mRNA expression were quantified by RT‐PCR, *n* = 4 (one‐way ANOVA followed by Tukey's multiple comparisons test). β‐actin is the protein loading control. Mean ± SEM, at least three independent experiments, *p < .05, **p < .01, ***p < .001, ****p < .0001 between indicated groups

Subsequently, we explored whether PCSK9 was involved in ox‐LDL‐induced myocardial apoptosis. Administration of evolocumab decreased the TUNEL‐positive nuclei number in the myocardium, the expression of apoptosis markers such as cleaved caspase‐3, cleaved caspase‐9 and Bax, while upregulated Bcl‐2 expression (Figure 2A–D). Consistently, in NMCMs, PCSK9 inhibition by two different approaches, pharmaceutic intervention by evolocumab or genetic intervention by shRNA adenovirus, reduced ox‐LDL‐induced cardiomyocyte apoptosis as evidenced by attenuated TUNEL staining and mitigated the related apoptotic signalling pathways (Figure 2E–L).

**FIGURE 2:**
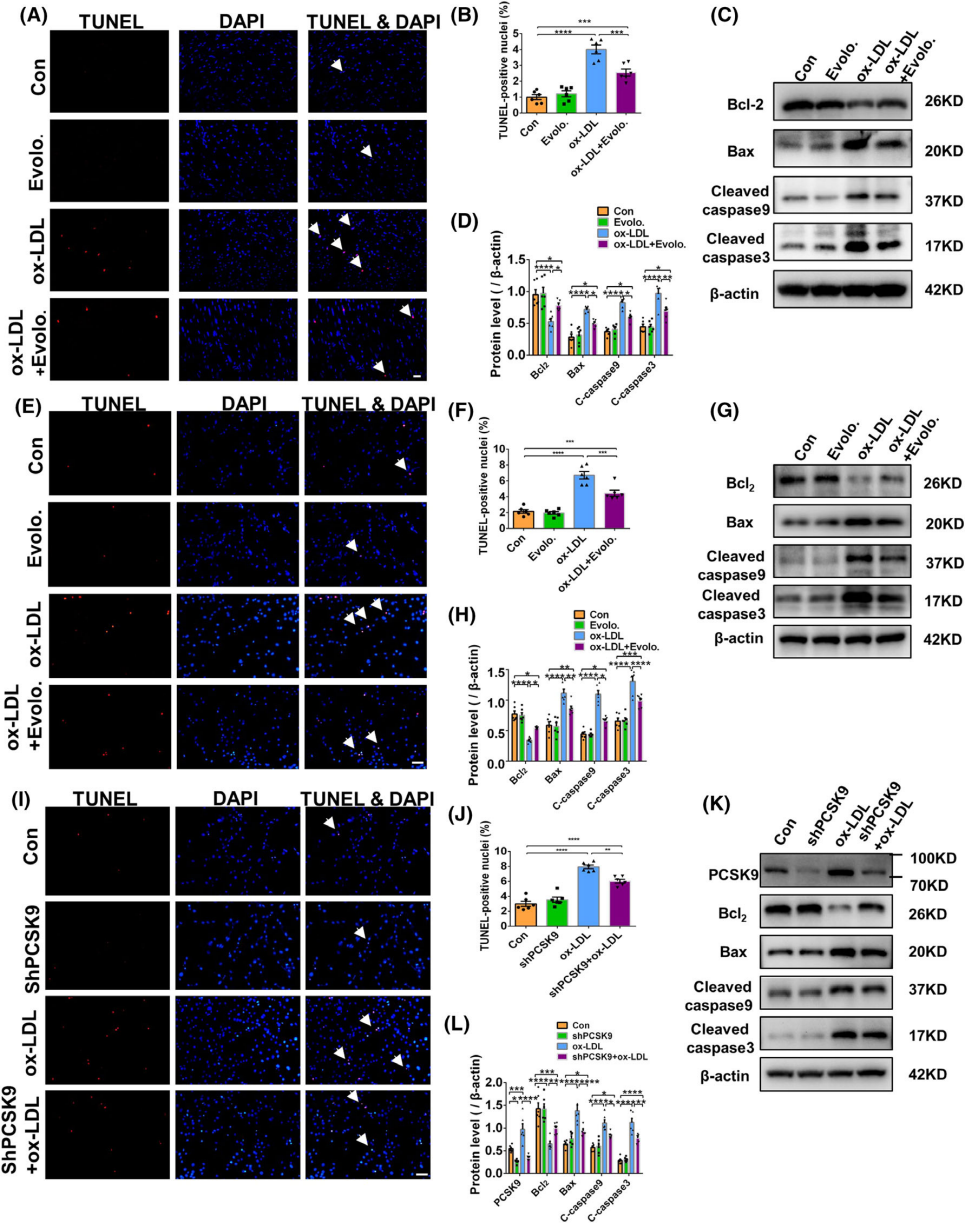
Attenuated apoptosis by evolocumab in oxidized low‐density lipoprotein (ox‐LDL)‐treated hearts and cultured primary cardiomyocytes. (A) Representative images of TUNEL‐stained and DAPI‐stained heart sections (arrows indicated TUNEL positive nuclei, scale bar: 20 μm). (B) TUNEL positive nuclei are quantified. More than 3000 nuclei per group were analysed. (C) Western blot analysis of Bcl‐2, Bax, cleaved caspase9 and cleaved caspase3 in heart tissue. The quantification is represented in (D). At least three independent experiments. (E) Representative images of TUNEL‐stained and DAPI‐stained neonatal mouse ventricular myocytes (NMVMs) (arrows indicate TUNEL positive nuclei, scale bar: 100 μm). (F) TUNEL positive nuclei are quantified. More than 3000 nuclei per group were analysed. (G) Protein expression of Bcl‐2, Bax, cleaved caspase9 and cleaved caspase3 in NMVMs. The quantification is represented (H). At least three independent experiments. (I) Representative images of TUNEL‐stained and DAPI‐stained NMVMs (arrows indicate TUNEL positive nuclei, scale bar: 100 μm). (J) TUNEL positive nuclei are quantified. More than 3000 nuclei per group were analysed. (K) Protein expression of PCSK9, Bcl‐2, Bax, cleaved caspase9 and cleaved caspase3 in NMVMs. The quantification is represented (L). At least three independent experiments. β‐Actin is the protein loading control. Mean ± SEM, *n* = 6 per group (one‐way ANOVA followed by Tukey's multiple comparisons test). *p < .05, **p < .01, ***p < .001, ****p < .0001 between indicated groups

The mitochondria constitute approximately 30% of the cardiomyocyte mass.^8^ The health of mitochondria is ensured by quality control mechanisms, including mitochondrial fission and fusion, while disruption of these processes leads to mitochondrial dysfunction and triggers oxidative stress and apoptosis.^9^ Therefore, we examined mitochondrial oxidative stress and fission in mice hearts. Our results showed that evolocumab counteracted the detrimental effects of ox‐LDL, as reflected by lower levels of myocardial ROS (Figure 3A) and less mitochondrial fission as demonstrated by the elevated mean size of mitochondria, reduced percentage of mitochondria less than 0.4 μm^2^ and elevated percentage of mitochondria more than 0.8 μm^2^ (Figure 3B,C). Evolocumab treatment attenuated ox‐LDL‐enhanced Drp1 phosphorylation at S616 and elevated ATP content in ox‐LDL‐treated hearts (Figure 3D,E). In agreement with our in vivo results, in cultured NMCMs and AMVMs incubated with ox‐LDL, mtROS levels and mitochondrial fragmentation degrees were markedly lower after treatment with evolocumab (Figure 3F–H,J). Meanwhile, the ox‐LDL induced Drp1 phosphorylation at S616 was markedly mitigated, and mitochondrial membrane potential and ATP content were increased in evolocumab‐treated NMCMs compared with vehicle‐treated NMCMs (Figure 3I,K). These results imply that inhibition of PCSK9 alleviates ox‐LDL‐induced mtROS and mitochondrial impairment in cardiomyocytes. Coherently, similar to the results obtained from evolocumab‐treated NMCMs, NMCMs treated with PCSK9 shRNA adenovirus also showed improved mitochondrial function (Figure S5).

**FIGURE 3:**
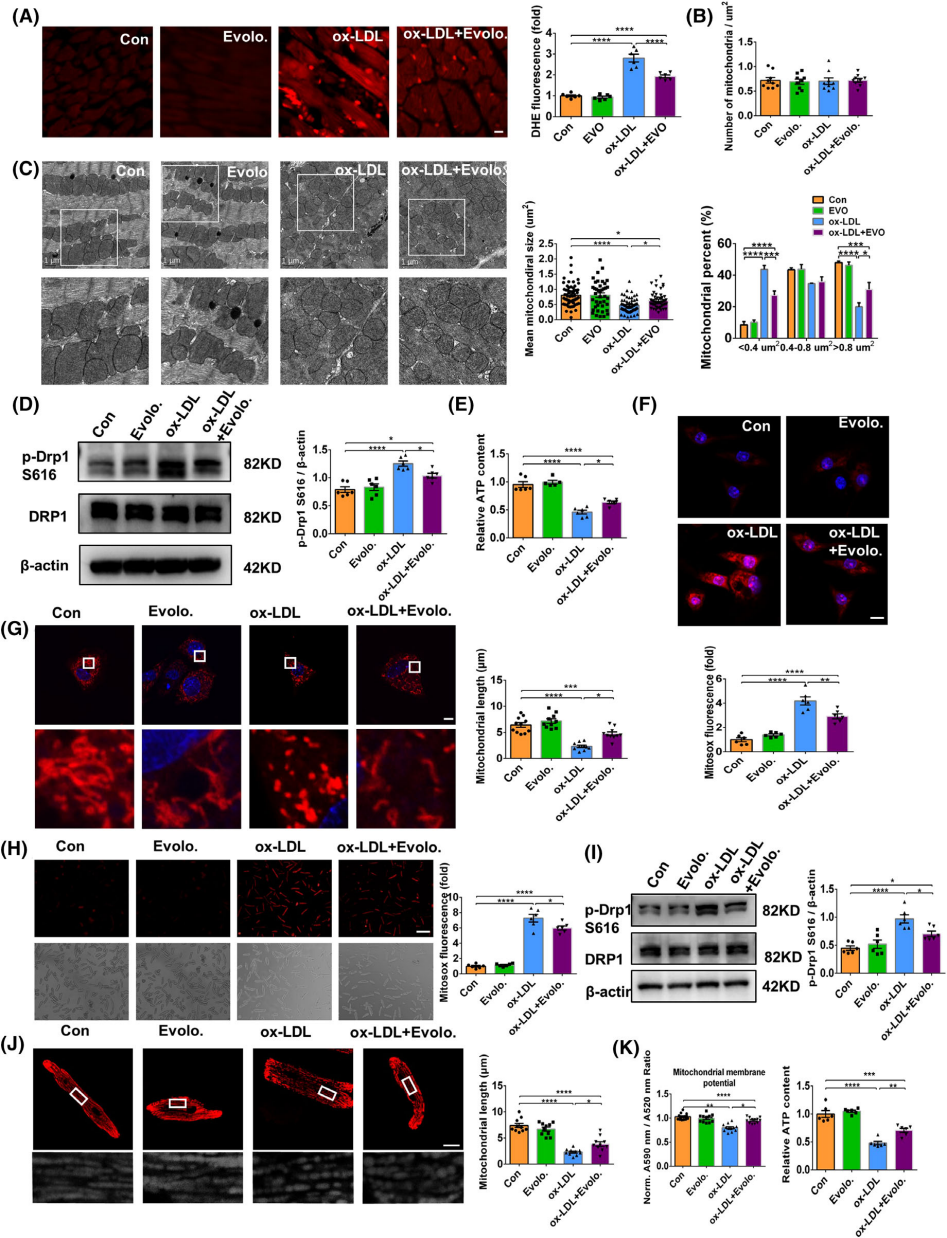
PCSK9 inhibition by evolocumab attenuated oxidized low‐density lipoprotein (ox‐LDL)‐induced mitochondrial dysfunction. (A) DHE staining (scale bar: 10 μm). DHE fluorescence intensity is quantified, *n* = 6 images/mouse, 3 mice/group. (B–C) Representative TEM images of cardiac mitochondrial morphology (scale bar: 1.0 μm). Quantitative analysis of mitochondria number, mean size of mitochondria and percentage of mitochondria classified into three categories by size, *n* = 9 images/mouse, 3 mice/group. (D) Protein expression of p‐Drp1 S616 and Drp1 in heart tissue. The quantification is represented in bar graphs (right panel). (E) Relative ATP content in heart tissue. (F) Representative images of neonatal mouse ventricular myocytes (NMVMs) dyed with MitoSOX(red) (scale bar: 10 μm). Average MitoSOX intensity in mitochondria of NMVMs is quantified (below panel), *n* = 18 images/group. (G) Representative images of NMVMs representing mitochondrial morphology dyed by MitoTracker Red (left panel, scale bar: 10 μm). Quantification of mitochondrial length of NMVMs is shown (right panel). More than 30 cells were assessed. (H) Representative images of MitoSOX fluorescence in AMVMs after ox‐LDL treatment (scale bar: 100 μm). Quantification of MitoSOX fluorescence intensity is shown, *n* = 18 images/ group. (I) Protein expression of p‐Drp1 S616 and Drp1 in NMVMs. The quantification is represented by bar graphs. (J) Representative images of AMVMs dyed by MitoTracker Red (scale bar: 10 μm). Mitochondrial length of AMVMs is quantified. More than 30 cells were assessed. (K) Analysis of mitochondrial membrane potential. NMVMs were stained with JC‐1 and fluorescence intensity was measured with a plate reader. The ratio of red/green fluorescence is shown. Relative ATP content in NMVMs after ox‐LDL and evolocumab treatment. β‐Actin is the protein loading control. Mean ± SEM, *n* = 6 (one‐way ANOVA followed by Tukey's multiple comparisons tests), at least three independent experiments, *p < .05, **p < .01, ***p < .001, ****p < .0001 between indicated groups

Since LOX‐1, a main ox‐LDL receptor, is recently found that it regulates PCSK9 in vascular tissues,^10^ we postulate that it mediates the synergistic effects of ox‐LDL and PCSK9 in the heart. In ox‐LDL‐treated mouse hearts, the expression of LOX‐1 was upregulated (Figure S6A). We then knockdown LOX‐1 in mouse hearts by adeno‐associated virus serotype 9 (AAV9) (Figure S6B,C). As expected, AAV9‐shLOX‐1 not only notably mitigated ox‐LDL‐induced LOX‐1 expression, but also reduced PCSK9 expression in vivo (Figure 4F; Figure S6D). In line with that effect, knockdown of LOX‐1 diminished cardiac hypertrophy induced by ox‐LDL, as demonstrated by reduced LV internal dimension, wall thickness (Figure 4A), HW/BW (Figure 4C), cardiomyocyte size (Figure 4D), mRNA expression of ANP, BNP and β‐MHC and fibrosis (Figure S6E–F, Figure 4E). Consistently, knockdown of LOX‐1 improved cardiac function in ox‐LDL‐treated mice as indicated by increased LVEF (Figure 4A), max dP/dt, and min dP/dt (Figure 4B). Moreover, knockdown of LOX‐1 also alleviated myocardial apoptosis, mitochondrial oxidative stress and fission in mice treated with ox‐LDL (Figures S6G–J and S7). These results collectively suggest that LOX‐1 is involved in ox‐LDL‐induced increases in PCSK9 expression, mitochondrial oxidative stress and fission, cardiomyocyte apoptosis and cardiac injuries.

**FIGURE 4:**
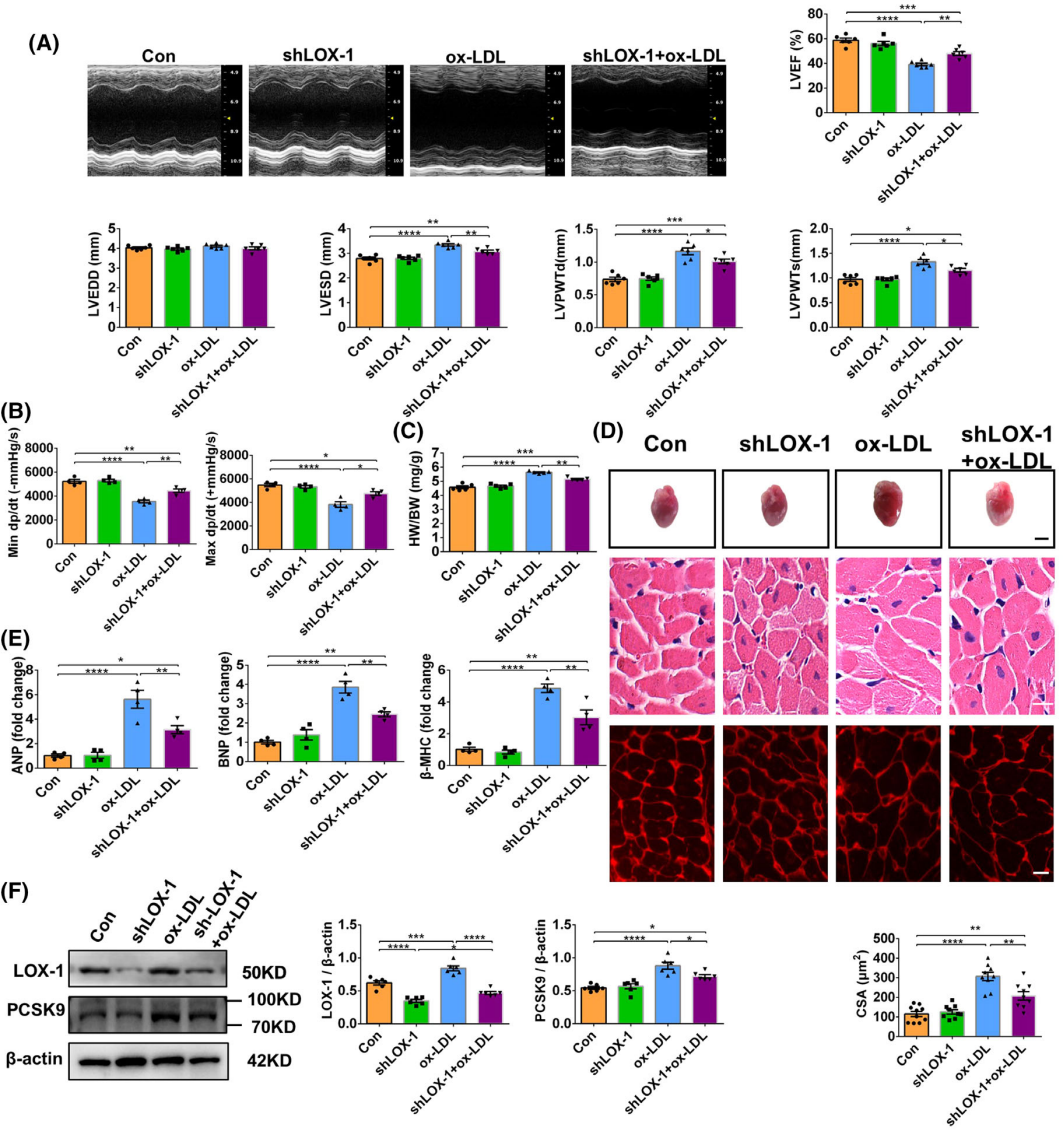
Role of LOX‐1 in cardiac function and PCSK9 expression. (A) Representative M‐mode echocardiography. And LVEF, LVEDD, LVESD, LVPWTd and LVPWTs were analysed by echocardiography. (B) Maximal +dp/dt and minimal –dp/dt. (C) Heart weight and body weight ratio (HW/BW). (D) Gross morphology and histological analysis of heart tissues. Upper panel: gross morphology, scale bars, 6.0 mm; middle panel: HE staining, scale bars, 20 μm; lower panel: WGA staining, scale bars, 10 μm. Cross‐sectional area (CSA) of cardiomyocytes, *n* = 25–30/mouse, 3 mice/group. (E) *ANP*, *BNP* and *β‐MHC* gene expression was quantified by RT‐PCR. (F) Protein expression of LOX‐1 and PCSK9 in heart tissue. The quantification is represented. β‐Actin is the protein loading control. Mean ± SEM, *n* = 4–6 (one‐way ANOVA followed by Tukey's multiple comparisons tests), at least three independent experiments, *p < .05, **p < .01, ***p < .001, ****p < .0001 between indicated groups

Although PCSK9 is produced mainly in livers, our study indicates that cardiomyocyte is also an important source of PCSK9 during ox‐LDL treatment, for increased PCSK9 expression in cardiomyocytes is found in vitro and in vivo when ox‐LDL is applied. Our results suggest that the increase in serum concentration of PCSK9, at least in part, owes to the heart. However, ox‐LDL can cause oxidative stress to other organs in addition to the heart, we therefore, cannot preclude the possibility that the liver also considerably causes the increase in serum concentration of PCSK9, especially considering the liver function is not taken into account in this study. Another limitation is, considering flow cytometry assessment is unavailable for cultured primary neonatal and adult cardiomyocytes, we used fluorescence quantification to quantify ROS (DHE, MitoSOX). Although this method is widely applied, it should be noteworthy that the subjective assessment using it may occur and lead to bias, thus other relevant methods such as quantifying protein carbonyls in isolated mitochondria, are warranted to corroborate our findings in future studies.

In summary, this study reports the effects of ox‐LDL on cardiac remodelling through PCSK9 for the first time. Ox‐LDL increases PCSK9 expression, mitochondrial oxidative stress, mitochondrial fission and cardiomyocyte apoptosis, leading to cardiac hypertrophy and dysfunction, while PCSK9 inhibition by pharmaceutical or genetic manners markedly alleviated the deleterious effects of ox‐LDL on cardiomyocytes. In addition, LOX‐1 mediates the bidirectional interaction between ox‐LDL and PCSK9. The cardioprotection induced by PCSK9 inhibition provides support to the potential of PCSK9 as a new target for cardiac hypertrophy and associated heart failure therapy.

## CONFLICT OF INTEREST

The authors declare no conflict of interest.

## Supporting information



Supporting information

Supporting information

Supporting information

Supporting information

Supporting information

Supporting information

Supporting information

Supporting information
