# Supplementary material for: PCSK9 participates in oxidized‐low density lipoprotein‐induced myocardial injury through mitochondrial oxidative stress and Drp1‐mediated mitochondrial fission
Source: Clin Transl Med. 2022 Feb 20;12(2):e729. doi: 10.1002/ctm2.729 (PMC8858617; doi:10.1002/ctm2.729)
Supplement: Supplementary file 1 — Supporting information [file CTM2-12-e729-s004.docx]

**SUPPLEMENTARY MATERIAL**

**PCSK9 participates in oxidized-low density lipoprotein-induced myocardial injury through mitochondrial oxidative stress and Drp1-mediated mitochondrial fission**

Xuan Li^†^, Fangjie Dai^†^, Hao Wang^†^, Ge Wei, Qiu Jiang, Peipei Yin, Shijun Wang, Junbo Ge, Cheng Yang^*^, Jian Wu^*^, Yunzeng Zou^*^

*** Corresponding authors. Email: zou.yunzeng@zs-hospital.sh.cn, wu.jian@zs-hospital.sh.cn. or yang.cheng@zs-hospital.sh.cn**

**This file includes:**

**Materials and methods**

**Supplementary Figure 1-7**

**Supplementary Table 1-3**

**1. Materials and methods**

**1.1 Collection of human serum samples**

A total of 33 individuals visiting Zhongshan Hospital were recruited in this study. Ten of them were clinically diagnosed with hyperlipidemia (HL) only, while 13 of them were diagnosed with both HL and heart failure (HF). The remaining 10 were apparently healthy people serving as controls. The demographic and clinical information of these participants was listed in Table S1. Their serum samples were collected and the serum levels of ox-LDL and PCSK9 were quantified by ELISA. This study was approved by the Ethics Committee of Zhongshan Hospital and performed according to the tenets of the Declaration of Helsinki. All patients signed an informed consent.

**1.2 Mice and treatments**

Adult male C57BL/6J mice (8–10 weeks old, 22–25 g) obtained from JieSiJie Laboratory Animal Co.,Ltd. (Shanghai, China) were subjected to ox-LDL (1.8 mg/kg/d in PBS, Yiyuan biotechnology, Guangzhou,China) or vehicle treatment in an equal volume by intraperitoneal injection for 4 weeks. To investigate the role of PCSK9 in ox-LDL-induced myocardial injury, evolocumab (a monoclonal antibody that inhibits human PCSK9, Repatha, Amgen), or vehicle were administered subcutaneously (10 mg/kg in PBS) every 7 days for 4 weeks to mice. The myocardial LOX-1 was knockdown in vivo by using an adeno-associated virus type 9 (AAV9) vector (AAV9-LOX-1) (pHBAAV-U6-MCS-LOX-1 shRNA-EGFP, Hanbio Co., Ltd., Shanghai, China). A total of 1 × 10^11^ viral particles (vp) of AAV9-LOX-1 or vector virus AAV9-EGFP (pHBAAV-U6-MCS-CMV-EGFP, Hanbio Co., Ltd., Shanghai, China) were subjected to mice by tail vein injection three weeks before ox-LDL treatment.

All animal experimental protocols were reviewed and approved by the Animal Care and Use Committee of of Zhongshan Hospital, Fudan University and were in compliance with the Guide for the Care and Use of Laboratory Animals published by the US National Institutes of Health (revised in 1996).

**1.3 Echocardiography and hemodynamic assessments**

M-mode echocardiography was performed using a Vevo 2100 high-frequency ultrasound system with a 30 MHz scanner (VisualSonics, Toronto, ON, Canada). Mice were anesthetized with isoflurane (1%–2%) to maintain heart rate at about 500 beats/minute. Left ventricular (LV) ejection fraction (LVEF), LV end-diastolic dimension (LVEDD), Left ventricular end-systolic dimension (LVESD), LV posterior wall end-diastolic thickness (LVPWTd), and LV posterior wall end-systolic thickness (LVPWTs) were acquired.

Invasive LV hemodynamic assessment was performed using the Power Laboratory system (AD Instruments, Castle Hill, NSW, Australia) connected with a micromanometric catheter (Millar 1.4-Fr, SPR 835, Millar Instruments, Houston, TX). The catheter was inserted into the left ventricle through the right common carotid artery. The following indexes were measured: left ventricular end-systolic pressure (LVESP), left ventricular end-diastolic pressure (LVEDP), the maximal rate of pressure rising (max dP/dt) and maximal rate of pressure fall (min dP/dt).

**1.4 Histological analysis**

After the hemodynamic analysis, mice were humanely euthanized by cervical dislocation, and hearts were harvested, the ratio of heart weight/body weight (HW/BW) was calculated. The hearts were fixed with 4% paraformaldehyde, embedded in paraffin using a standard protocol, cut into 5 μm thick sections, and stained with hematoxyline-eosin (H&E) to observe the morphology of cardiomyocytes, or with Masson’s trichrome to measure the degree of cardiac fibrosis. Texas RedTM-X-conjugated wheat germ agglutinin (WGA) (Invotrogen) was employed to observe cell membranes to further evaluate cardiomyocyte hypertrophy.

**1.5 Transmission electron microscopy**

Samples from the LV tissues were obtained (≤1 mm^3^) and fixed in phosphate buffer (PB, 0.1 M, pH 7.4) containing 4% formaldehyde and 2.5% glutaraldehyde for 12 h at 4^o^C. Samples were then washed three times with PB buffer, fixed with 1% OsO_4_ for 30 minutes, and contrasted in 1% uranyl acetate in ddH_2_O overnight. After being treated in ethanol on ice with gradient dehydration, samples were infiltrated with Epon812 resin (Electron microscopy sciences), polymerized at 60^o^C for 48 hours, cut into ultrathin sections (70 nm) on a Leica ultramicrotome, and examined under transmission electron microscope (JEM-1230, JEOL Ltd., Tokyo, Japan) at 80 kV. Images were taken with a Gatan 2048x2048 CCD camera (Orius 830 camera) and captured by a technician blinded to the treatment. At least 9 fields were randomly selected for analyzing mitochondrial size and number with Image J software. The percentage of mitochondria sorted into three categories by size (<0.4 μm^2^, within 0.4-0.8 μm^2^, >0.8 μm^2^) was counted as described previously^1, 2^.

**1.6 Preparation and culture of neonatal and adult** **mouse cardiomyocytes**

Neonatal mouse ventricular myocytes (NMVMs) were isolated from 1-3-day-old mice. After mice were disinfected with 75% ethanol, hearts were removed aseptically, rinsed and trimmed in a dish filled with phosphate-buffered saline (PBS), cut into around 1mm^3^ pieces, and then washed with PBS 3 times. Heart pieces were digested with 0.08% collagenase 1 (Worthington, USA) at 37°C for 3 minutes and neutralized with complete medium (DMEM/F12 culture medium supplemented with 10% fetal bovine serum). The above steps were repeated until the fragments were digested completely. Cells were then collected by centrifuging the cell suspension at 1000 rpm for 5 minutes, resuspended in complete medium and plated for 1-2 h. Subsequently, the suspended cells were collected and plated in culture dishes. After 24 hours, cardiomyocytes were starved with serum-free medium for 12 hours, then ox-LDL (100 ug/ml), evolocumab (500ug/ml), or a combination of both were added to the fresh serum-free medium and cells were incubated at 37°C for another 24 hours. For adenoviral infection, cardiomyocytes were infected by PCSK9 shRNA adenovirus for 8h after being plated for 24 hours, then cultured for 24 hours followed by being starved for 12 hours and incubated with ox-LDL (100 ug/ml) under serum-free condition for 24 hours. The PCSK9 shRNA adenovirus (pADV-U6-shRNA (Pcsk9)-CMV-MCS) and negative control (Ad-control) were purchased from Obio Technology (Shanghai, China).
 Adult mouse ventricular myocytes (AMVMs) were isolated from mice as described previously ^3^. After the mouse was anesthetized, the mouse's chest was wiped with 75% ethanol and cut through to expose the heart. The descending aorta and inferior vena cava were cut off and EDTA buffer was injected into the bottom of the right ventricle (RV) steadily. After the aorta was clamped, the heart was excised through the incision, and EDTA buffer was injected from the apical area into the left ventricle. Perfusion buffer, followed by collagenase buffer were injected into the left ventricle (LV) through the previous hole. After the digestion was complete, the tissue was cut gently into small pieces and dissociated. After that, the enzyme reaction was inhibited with the Stop solution and the cell suspension was passed through a filter with 100 μm pore size. Cardiomyocytes were collected by gravity settlement for 20 minutes, and used for culture after re-introduction of calcium. Pure cardiomyocytes were resuspended in plating medium (Medium 199, supplemented with 5% FBS, 2,3-Butanedione monoxime and 1% Penicillin-Streptomycin), and then plated on culture dishes pre-coated with laminin (5 μg/ml, Thermo Scientific). One hour later, the plating medium was changed to the culture medium (Medium 199, supplemented with 5% (w/v）bovine serum albumin, ITS supplement, 2,3-Butanedione monoxime, chemically defined lipid concentrated and 1% Penicillin-Streptomycin) and ox-LDL (100 ug/ml), evolocumab (500ug/ml), or a combination of both were added to incubate with cells for 24 hours.

**1.7 Apoptosis analysis**

Heart paraffin sections and cultured cardiomyocytes were stained with TUNEL (terminal deoxynucleotidal transferase-mediated dUTP nick end-labelling) Apoptosis Assay Kit (Beyotime, C1089, China) according to manufacturer’s instructions, then examined under a fluorescence microscope (Olympus, Tokyo, Japan). Six random fields were elected for observation in a blinded manner.

**1.8 Assessment of mitochondrial function**

An ATP (adenosine triphosphate) assay kit (Beyotime, S0027, China) was used for ATP content measurement of heart tissue or cultured cardiomyocytes, a mitochondrial membrane potential assay kit with JC-1 (Beyotime) was used for mitochondrial membrane potential detection of cultured cardiomyocytes, a Mito-Tracker Red CMXRos probe (Beyotime) was used for mitochondrial morphology evaluation of neonatal and adult mouse cardiomyocytes (the captured images by focal microscope were analyzed by ImageJ software to assess the average mitochondrial length^4^), Dihydroethidium (DHE) probe (Beyotime) was utilized for detection of In situ superoxide production of fresh heart tissues, and MitoSOX™ Red Mitochondrial Superoxide Indicator (ThermoFisher) was utilized for detection of mitochondrial ROS levels of cultured neonatal and adult mouse cardiomyocytes. All assays were carried out following the manufacturer's instructions.

**1.9 Quantitative real-time polymerase chain reaction**

Total RNA was extracted from heart tissues and cultured cardiomyocytes using a total RNA isolation kit (Sangon Biotech, China) in accordance with the manufacturer’s instructions. A total of 500ng purified RNA was conversed to cDNA using TaKaRa PrimeScript™ Reverse Transcription Master mix. Then Real-time quantitative PCRs were performed using TaKaRa TB Green^®^ Premix Ex Taq™. The target gene expression was normalized to Graph using the 2^-ΔΔCT^ method. The primers used are listed in Table S2.

**1.10 Western blotting**

The ventricular tissues or cultured cardiomyocytes were lysed with RIPA lysis buffer (Beyotime, P0013B, China) containing a mixture of protease inhibitors and phosphatase inhibitors. Western blotting was performed according to the standard procedure as we described previously^5^. The immunoblotting images were captured with ChemiDoc™ Imaging System (Bio-Rad, CA, US). The commercially available antibodies used in this study were shown in Table S3.

**1.11 ELISA**

The ox-LDL and PCSK9 levels in human blood samples, mice serum or cardiomyocyte supernatants were measured using commercially available kits (Human oxidized low density lipoprotein (OxLDL) ELISA Kit, NG-EA1584; Human Proprotein Convertase Subtilisin exin Type 9 (PCSK9) ELISA Kit, NG-EA1134; Mouse Proprotein Convertase Subtilisin Kexin type 9 (PCSK9) ELISA Kit, NG-EB1804; Mouse oxidized lowdensity lipoprotein (OxLDL) ELISA Kit, NG-EB968; Shanghai Yuanmin Biotechnology Co., Ltd; www.newgeorge.cn) according to the manufacturer’s instruction.

**1.12 Statistical analysis**

GraphPad Prism 8 (GraphPad Software, Version 8.01, San Diego, CA, United States) was used to perform statistical analysis. All measurement data were represented as mean ± SE. The normality tests were determined by the Shapiro–Wilk test. Comparisons among groups were analyzed with Student’s t-test (within 2 groups) or one-way ANOVA with post hoc comparisons by Tukey’s multiple comparisons test (among 3 or 4 groups) based on the distribution of data. *p*-values < 0.05 are considered statistically significant.

**Reference**

1. Wang JX, Jiao JQ, Li Q, Long B, Wang K, Liu JP, Li YR and Li PF. miR-499 regulates mitochondrial dynamics by targeting calcineurin and dynamin-related protein-1. *Nat Med*. 2011;17:71-8.

2. Ding M, Feng N, Tang D, Feng J, Li Z, Jia M, Liu Z, Gu X, Wang Y, Fu F and Pei J. Melatonin prevents Drp1-mediated mitochondrial fission in diabetic hearts through SIRT1-PGC1alpha pathway. *J Pineal Res*. 2018;65:e12491.

3. Ackers-Johnson M, Li PY, Holmes AP, O'Brien SM, Pavlovic D and Foo RS. A Simplified, Langendorff-Free Method for Concomitant Isolation of Viable Cardiac Myocytes and Nonmyocytes From the Adult Mouse Heart. *Circ Res*. 2016;119:909-20.

4. Ren J, Sun M, Zhou H, Ajoolabady A, Zhou Y, Tao J, Sowers JR and Zhang Y. FUNDC1 interacts with FBXL2 to govern mitochondrial integrity and cardiac function through an IP3R3-dependent manner in obesity. *Sci Adv*. 2020;6.

5. Dai F, Li X, Li X, Ding Z, Xu R, Yin P, Wang S, Ge J, Wu J and Zou Y. Caspase-1 Abrogates the Salutary Effects of Hypertrophic Preconditioning in Pressure Overload Hearts via IL-1beta and IL-18. *Front Mol Biosci*. 2021;8:641585.


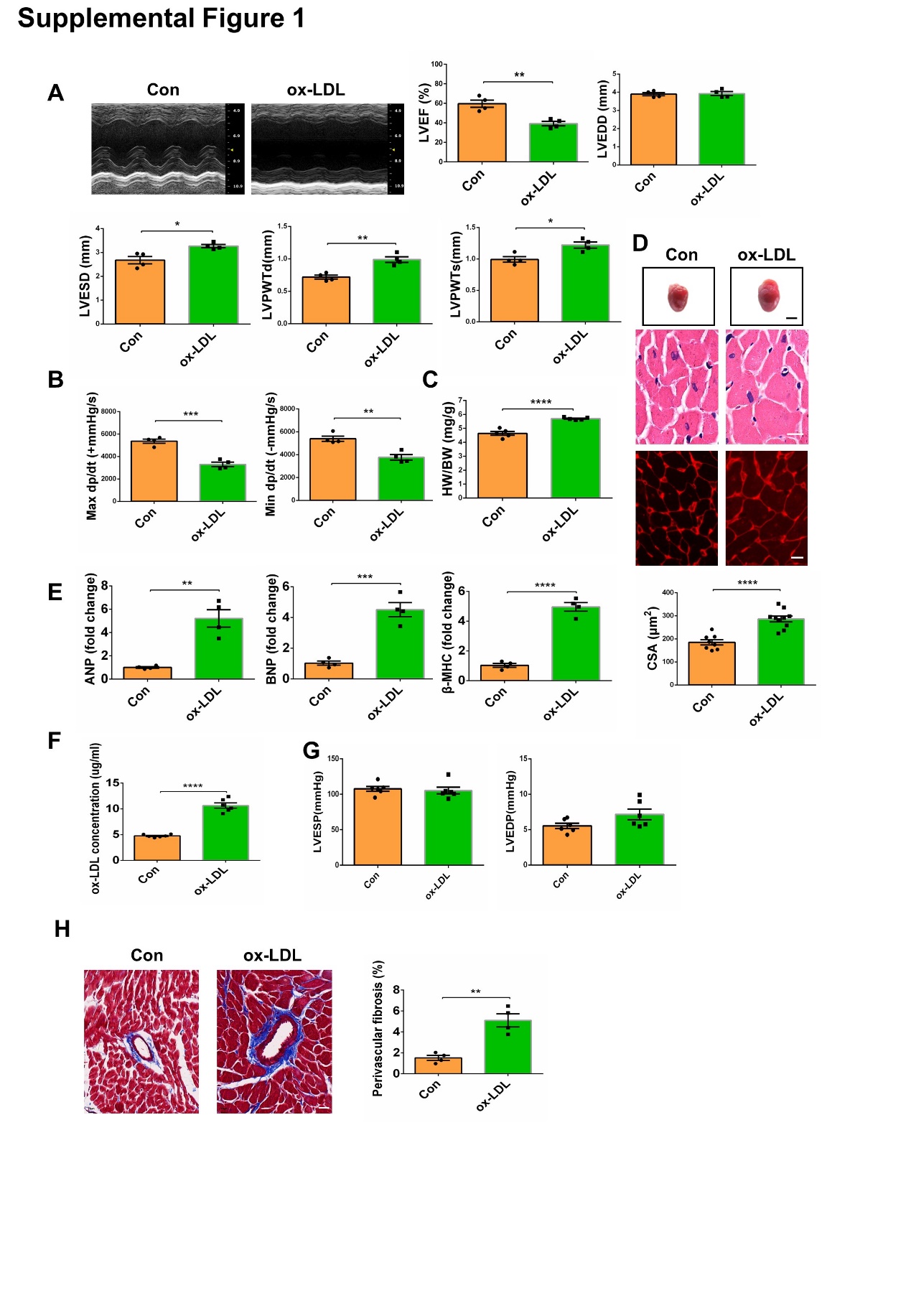


**Fig.S1 Cardiac hypertrophy and dysfunction in the hearts subjected to ox-LDL treatment. (A)** Representative left ventricular (LV) M-mode echocardiography. LV ejection fraction (LVEF), LV end-diastolic dimension (LVEDD), Left ventricular end-systolic dimension (LVESD), LV posterior wall end-diastolic thickness (LVPWTd), and LV posterior wall end-systolic thickness (LVPWTs) were analyzed by echocardiography. **(B)** Maximal dp/dt and minimal dp/dt were determined by hemodynamic analysis. **(C)** Heart weight and body weight ratio (HW/BW). **(D)** Cross-sectional area (CSA) of cardiomyocytes. n=25-30/mouse, 3 mice/group. Gross morphology and histological analysis of heart tissues. Upper panel: gross morphology, scale bars, 6.0 mm; middle panel: HE staining, scale bars, 20 μm; lower panel: WGA staining, scale bars, 10 μm. **(E)** *ANP***,** *BNP* and *β-MHC* mRNA expression were quantified by RT-PCR. At least 3 independent experiments. **(F)** Serum levels of ox-LDL in ox-LDL-treated mice by ELISA. **(G)** Left ventricular end-systolic pressure (LVESP), left ventricular end-diastolic pressure (LVEDP) were determined by hemodynamic analysis. **(H)** Perivascular fibrosis stained by Masson’s trichrome. Scale bar, 20 μm. The quantification is shown (right panel). n=4/mouse. Mean ± SEM; n = 4 to 6 mice per group (Student’s t-test), **p* < 0.05, ***p* < 0.01, ****p* < 0.001, **** *p* < 0.0001 between indicated groups.


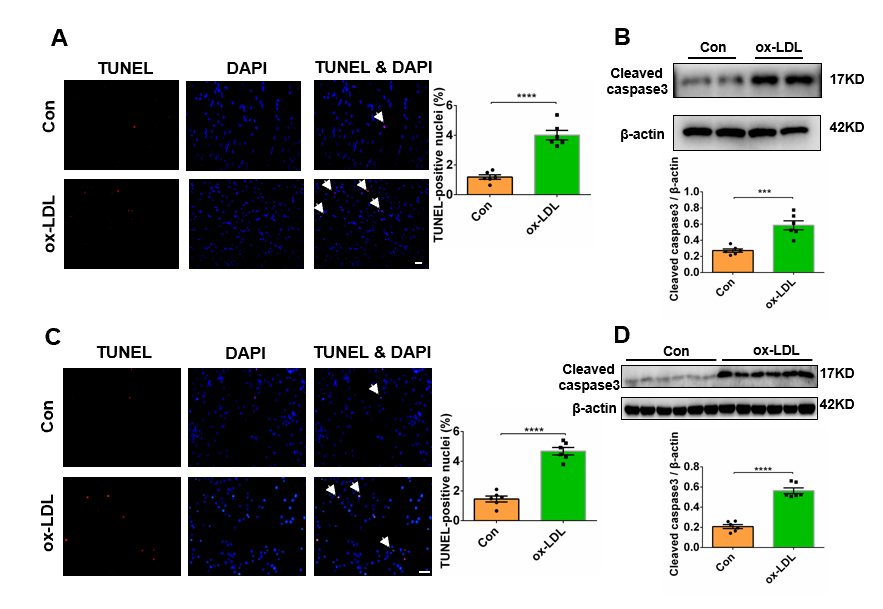


**Fig. S2** **Apoptosis in ox-LDL-treated hearts and cultured primary cardiomyocytes. (A)** TUNEL assay by double staining with DAPI (blue) and TUNEL (red) examined elevated apoptosis in ox-LDL-treated hearts (arrows indicate TUNEL positive nuclei, scale bar: 20 μm). The quantification of TUNEL positive nuclei is represented in bar graphs (right panel). More than 3000 nuclei were analyzed. **(B)** Western blot for active caspase-3 protein in ox-LDL-treated hearts. The quantification is shown. **(C)** TUNEL assay by double staining with DAPI (blue) and TUNEL (red) of cultured neonatal mouse ventricular myocytes (NMVMs) (arrows indicate TUNEL positive nuclei, scale bar: 100 μm). The quantification is represented in bar graphs (right panel). More than 3000 nuclei were analyzed. **(D)** Western blot for active caspase-3 protein in NMVMs treated with ox-LDL (100ug/ml) for 24 h; The quantification is shown. β-actin is the protein loading control. Mean ± SEM, n=6 per group (Student’s t-test), at least 3 independent experiments, ****p* < 0.001, *****p* < 0.0001 between indicated groups.


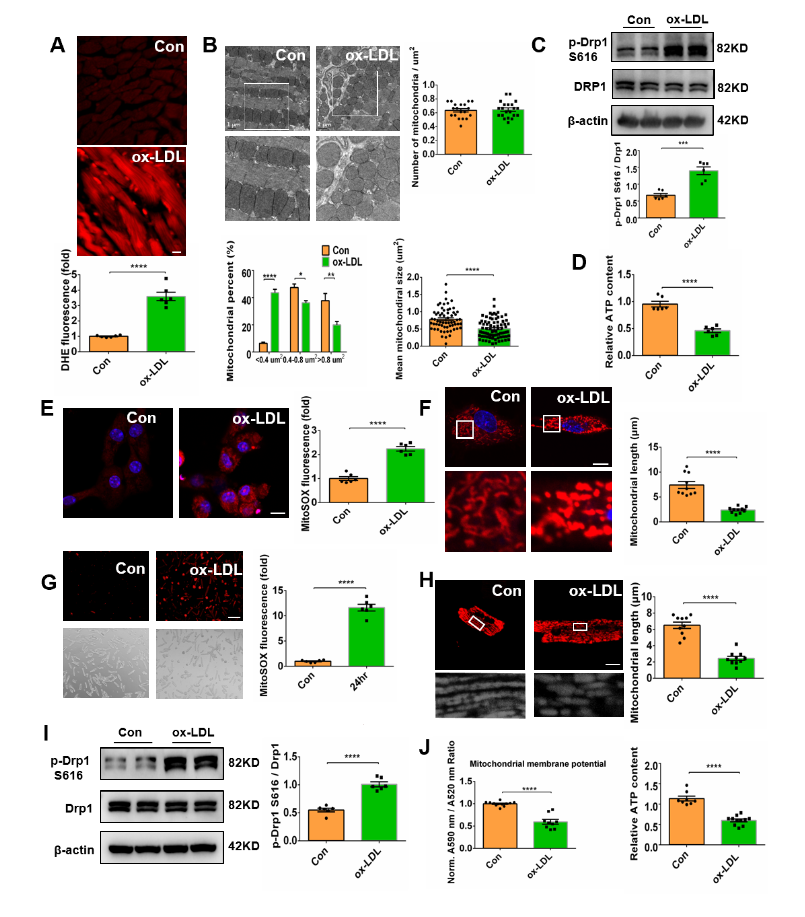


**Fig. S3 Mitochondrial dysfunction in ox-LDL treated hearts and cultured primary cardiomyocytes. (A)** DHE staining determined more ROS generation in ox-LDL-treated hearts (left panel, scale bar: 10 μm). Fluorescence intensity is quantified (right panel). n=6 images/mouse, 3 mice/group. **(B)** Representative transmission electron microscopic (TEM) images of cardiac mitochondrial morphology. Scale bar: 1.0 µm. Quantitative analysis of mitochondria number, mean size of mitochondria and percentage of mitochondria classified into three categories by size. n=9 images/mouse, 3 mice/group. **(C)** Western blot analysis of p-Drp1 S616 and Drp1 in ox-LDL-treated heart. The quantification is represented in bar graphs. **(D)** Relative mitochondrial ATP content in heart tissue. n = 6 hearts/group. **(E)** Representative confocal microscope images of NMVMs stained with the mitochondrial-specific reactive oxygen species indicator MitoSOX (red) (left panel, scale bar: 10 μm). Average MitoSOX intensity in mitochondria is quantified (right panel). n=18 images per group. **(F)** Representative confocal microscope images of NMVMs showing mitochondrial morphology stained by MitoTracker Red (left panel, scale bar: 10 μm). Quantification of mitochondrial length is shown in bar graphs (right panel). More than 30 cells were assessed. **(G)** MitoSOX fluorescence in adult mouse ventricular myocytes (AMVMs) after ox-LDL treatment (scale bar: 100 μm). Quantification of MitoSOX fluorescence intensity is shown. n=18 images per group. **(H)** Representative confocal microscope images of AMVMs stained by MitoTracker Red (scale bar: 10 μm). Mitochondrial length of AMVMs is quantified. More than 30 cells were assessed. **(I)** Western blot analysis of p-Drp1 S616 and Drp1 in NMVMs. The quantification is represented by bar graphs. **(J)** Analysis of mitochondrial membrane potential after ox-LDL treatment. NMVMs were stained with JC-1 and fluorescence intensity was measured with a plate reader. The ratio of red/green fluorescence is shown. Relative ATP content in NMVMs after ox-LDL treatment. β-actin is the protein loading control. Mean ± SEM, n=6 images or visual fields per group (Student’s t-test), at least 3 independent experiments, **p* < 0.05, ***p*< 0.01, *** *p* < 0.001, *****p*< 0.0001 between indicated groups.


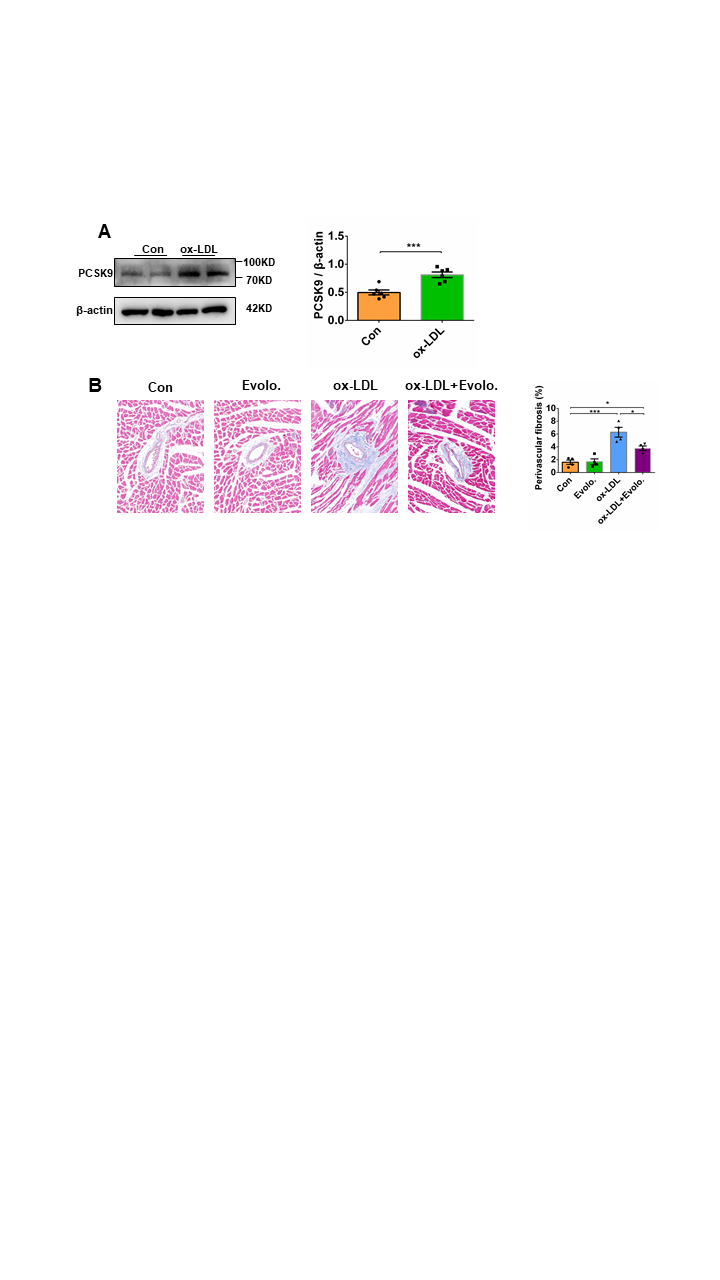
**Figure S4. PCSK9 expression and fibrosis. (A)** Western blot analysis of PCSK9 expression in ox-LDL-treated adult mouse ventricular myocytes (AMVMs). At least 3 independent experiments. n=6 (Student’s t-test). **(B)** Perivascular fibrosis stained by Masson’s trichrome. Scale bar, 30 μm. The quantification is shown (right panel). n=4/mouse, 4 mice/group. (one-way ANOVA followed by Tukey’s multiple comparisons test). β-actin is the protein loading control. Mean ± SEM, *p < 0.05, *** p < 0.001 between indicated groups.


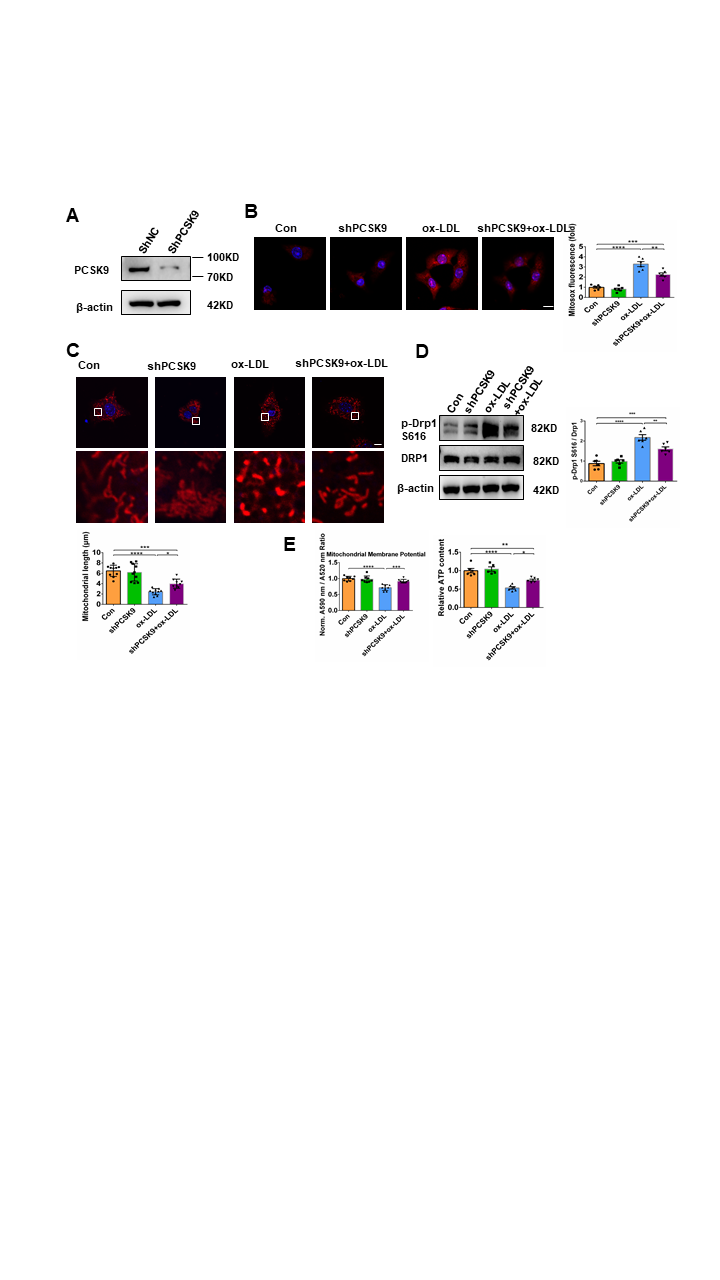
**Figure S5. PCSK9 inhibition by PCSK9 shRNA adenovirus infection alleviated ox-LDL-induced mitochondrial dysfunction. (A)** Representative images for PCSK9 shRNA knock down efficiency verification. shNC: cultured cardiomyocytes incubated with empty vector; shPCSK9: cultured cardiomyocytes incubated with PCSK9 shRNA. **(B)** MitoSOX fluorescence in NMVMs (left panel, scale bar: 10 μm). Average MitoSOX intensity in mitochondria is quantified (right panel). n=18 images per group. **(C)** MitoTracker fluorescence in NMVMs (scale bar: 10 μm). Mitochondrial length of AMVMs is quantified (below panel). More than 30 cells were assessed. **(D)** Western blot analysis of p-Drp1 S616 and Drp1 in NMVMs. The quantification is represented in bar graphs. **(E)** Analysis of mitochondrial membrane potential and relative ATP content in NMVMs after adenovirus infection and ox-LDL treatment. β-actin is the protein loading control. Mean ± SEM, n=6 (one-way ANOVA followed by Tukey’s multiple comparisons test), at least 3 independent experiments, **p* < 0.05, ***p* < 0.01, ****p* < 0.001, *****p* < 0.0001 between indicated groups.


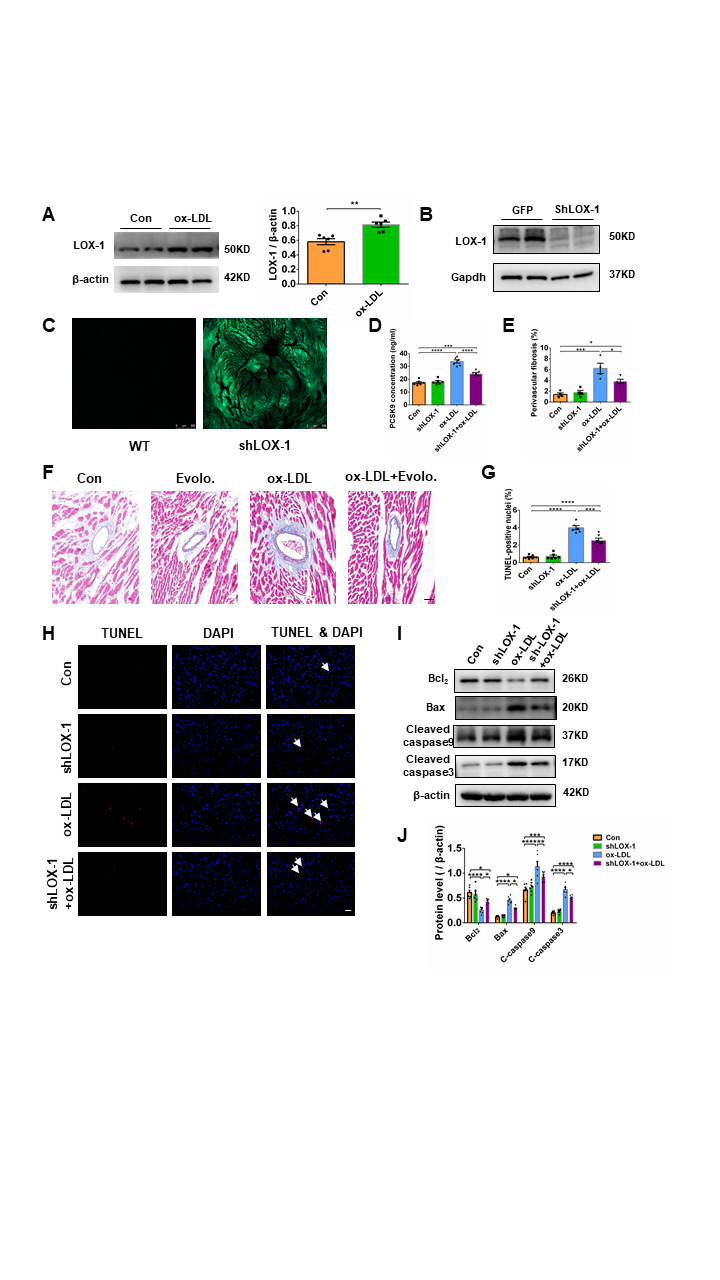


**Figure S6. LOX-1 expression, fibrosis and apoptosis. (A)** Western blot analysis of LOX-1 expression in ox-LDL-treated hearts. The quantification is shown (right panel). n=6 (Student’s t-test). **(B)** Western blot analysis of LOX-1 expression in heart tissues from mice injected with AAV9-shLOX-1. GAPDH is the protein loading control. **(C)** GFP fluorescence in heart sections from wild type mice and mice injected with AAV9-shLOX-1. **(D)** Serum levels of PCSK9 by ELISA. **(E-F)** Perivascular fibrosis stained by Masson’s trichrome. Scale bar, 25 μm. The quantification is shown **(E)** n=4/mouse. **(G-H)** Representative images of TUNEL-stained and DAPI-stained heart sections (arrows indicated TUNEL positive nuclei, scale bar: 100 μm). TUNEL positive nuclei are quantified **(G)**. More than 3000 nuclei were analyzed. **(I-J)** Western blot analysis of Bcl-2, Bax, Cleaved caspase9 and Cleaved caspase3 in heart tissue. The quantification is represented **(J)**. β-actin or GAPDH is the protein loading control. Mean ± SEM, n=4-6 (one-way ANOVA followed by Tukey’s multiple comparisons test), at least 3 independent experiments, **p* < 0.05, ***p* < 0.01, ****p* < 0.001, *****p* < 0.0001 between indicated groups.


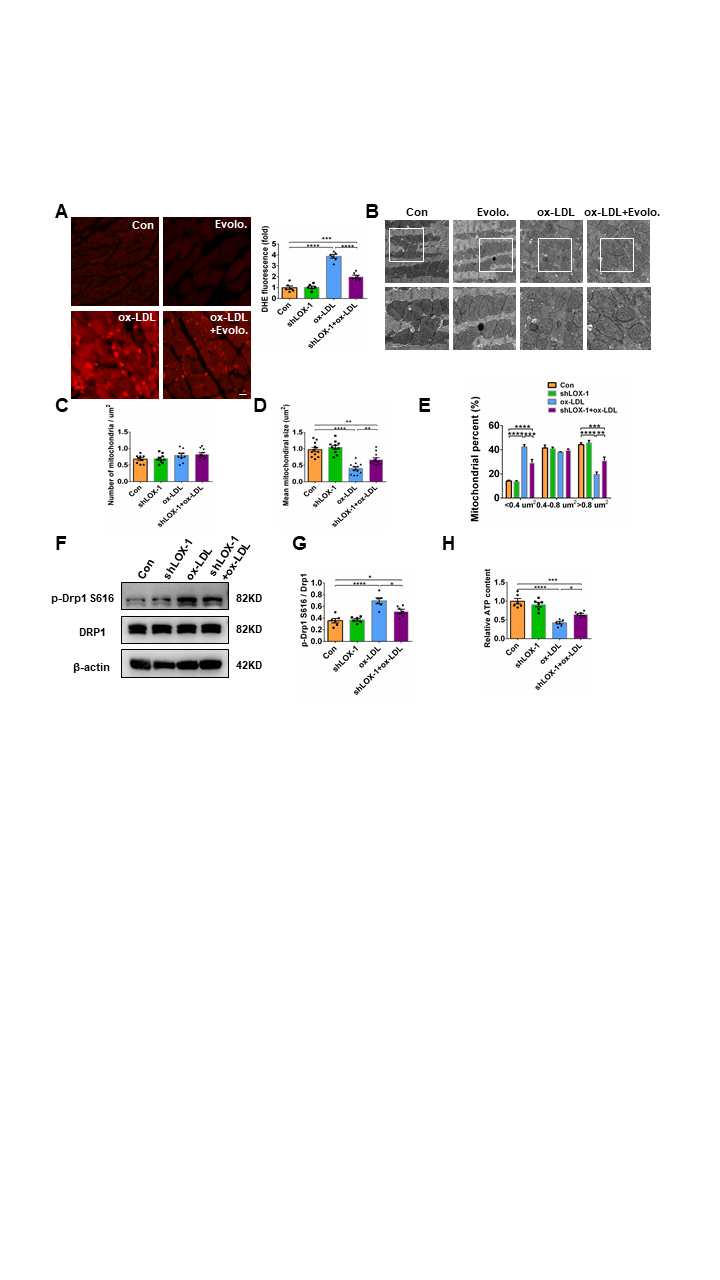
**Figure S7. Role of LOX-1 in oxidative stress and mitochondrial fission in ox-LDL-treated mice. (A)** DHE staining (scale bar: 10 μm). DHE fluorescence intensity is quantified (right panel). n=6 images/mouse, 3 mice/group. **(B-E)** Representative TEM images of cardiac mitochondrial morphology (Scale bar: 1.0 µm). Quantitative analysis of mitochondria number, mean size of mitochondria and percentage of mitochondria classified into three categories by size. n=9 images/mouse, 3 mice/group. **(F-G)** Western blot analysis of p-Drp1 S616 and Drp1 in heart tissue. The quantification is represented **(G)**. **(H)** Relative ATP content in heart tissue. n = 6 hearts/group. Mean ± SEM, n=6 (one-way ANOVA followed by Tukey’s multiple comparisons test), at least 3 independent experiments, **p* < 0.05, ***p* < 0.01, ****p* < 0.001, *****p* < 0.0001 between indicated groups.

**Table S1: Basic characteristic of human blood samples with hyperlipidemia and heart failure (HL+HF), hyperlipidemia (HL) or not (Con)**

Table S1. The demographic and clinical information of the patients

|  | Control | HL | HL+HF | *P* |
| --- | --- | --- | --- | --- |
|  | (n = 10) | (n = 10) | (n = 13) |  |
| **Age, year** | | | | |
|  | 56.00 (51.75, 61.75) | 58.50 (53.75, 63.75) | 69.00 (56.00, 75.50) | 0.0548 |
| **Gender, n** | | | | |
| Female | 3 | 5 | 3 | 0.3838 |
| Male | 7 | 5 | 10 |  |
| **ALT, U/L** | | | | |
|  | 26.50 (12.00, 36.25) | 27.00 (18.00, 38.00) | 21.00 (10.50, 27.00) | 0.2787 |
| **AST, U/L** | | | | |
|  | 21.50 (16.50, 26.25) | 26.00 (18.50, 32.00) | 20.00 (11.50, 23.00) | 0.1248 |
| **eGFR, mL/min/1.73m^2^** | | | | |
|  | 96.50 (89.00, 103.30) | 89.50 (84.75, 93.50) | 76.00 (63.00, 93.00) | 0.0144 |
| **TC, mmol/L** | | | | |
|  | 4.665 (3.498, 5.085) | 5.165 (4.803, 6.055) | 4.010 (3.455, 4.990) | 0.0465 |
| **TG, mmol/L** | | | | |
|  | 1.075 (0.905, 1.793) | 1.745 (1.213, 2.135) | 1.480 (1.200, 1.905) | 0.3301 |
| **LDL-C, mmol/L** | | | | |
|  | 2.660 (1.510, 3.018) | 3.025 (2.013, 4.080) | 2.220 (1.655, 3.220) | 0.2349 |

Data were median with quartiles. ALT, alanine transaminase; AST, aspartate transaminase; eGFR, estimated glomerular filtration rate; TC, total cholesterol; TG, triglycerides; LDL-C, low-density lipoprotein cholesterol.

**Table S2: Primers for quantitative Real-time PCR**

Table S2. All the primers used in the study

| Gene | Forward (5’ to 3’) | Reverse (5’ to 3’) |
| --- | --- | --- |
| Nppa | TTCGGGGGTAGGATTGACAG | CACACCACAAGGGCTTAGGA |
| Nppb | TGTTTCTGCTTTTCCTTTATCTG | TCTTTTTGGGTGTTCTTTTGTGA |
| Myh7 | TACTTGCTACCCTCAGGTGGCT | TGTCATCGGGCACAAAAACATC |
| Gapdh | TCACCATCTTCCAGGAGCGAGAC | TGAGCCCTTCCACAATGCCAAAG |

**Table S3:** **Primary antibodies used in the Western blot analysis**

Table S3. Primary antibodies for Western blot experiments

| **Antibody** | **Company** | **Catalog＃** | **Dilution ratio** |
| --- | --- | --- | --- |
| Phospho-DRP1(Ser616) Antibody | Cell Signaling Technology, BOSTON | 3455 | 1:1000 |
| DRP1(D6C7) Rabbit mAb | Cell Signaling Technology, BOSTON | 8570 | 1:1000 |
| Cleaved Caspase-3 (Asp175) Antibody | Cell Signaling Technology, BOSTON | 9661 | 1:1000 |
| Anti-PCSK9 antibody | Abcam, Cambridge | ab31762 | 1:1000 |
| Anti-Bcl-2 antibody | Abcam, Cambridge | ab196495 | 1:1000 |
| Bax Antibody | Cell Signaling Technology, BOSTON | 2772 | 1:1000 |
| Cleaved Caspase-9 (Asp353) Antibody (Mouse Specific) | Cell Signaling Technology, BOSTON | 9509 | 1:1000 |
| Rabbit Anti-LOX1 antibody | Biosynthesis Biotechnology, Beijing | Bs-2044R | 1:1000 |
| β-Actin mouse monoclonal Antibody | Weiao Biotechnology, Shanghai | WB0196 | 1:5000 |
| GAPDH mouse monoclonal Antibody | Weiao Biotechnology, Shanghai | WB0197 | 1:5000 |
