# Supplementary figures and images for: PCSK9 participates in oxidized‐low density lipoprotein‐induced myocardial injury through mitochondrial oxidative stress and Drp1‐mediated mitochondrial fission
Source: Clin Transl Med. 2022 Feb 20;12(2):e729. doi: 10.1002/ctm2.729 (PMC8858617; doi:10.1002/ctm2.729)

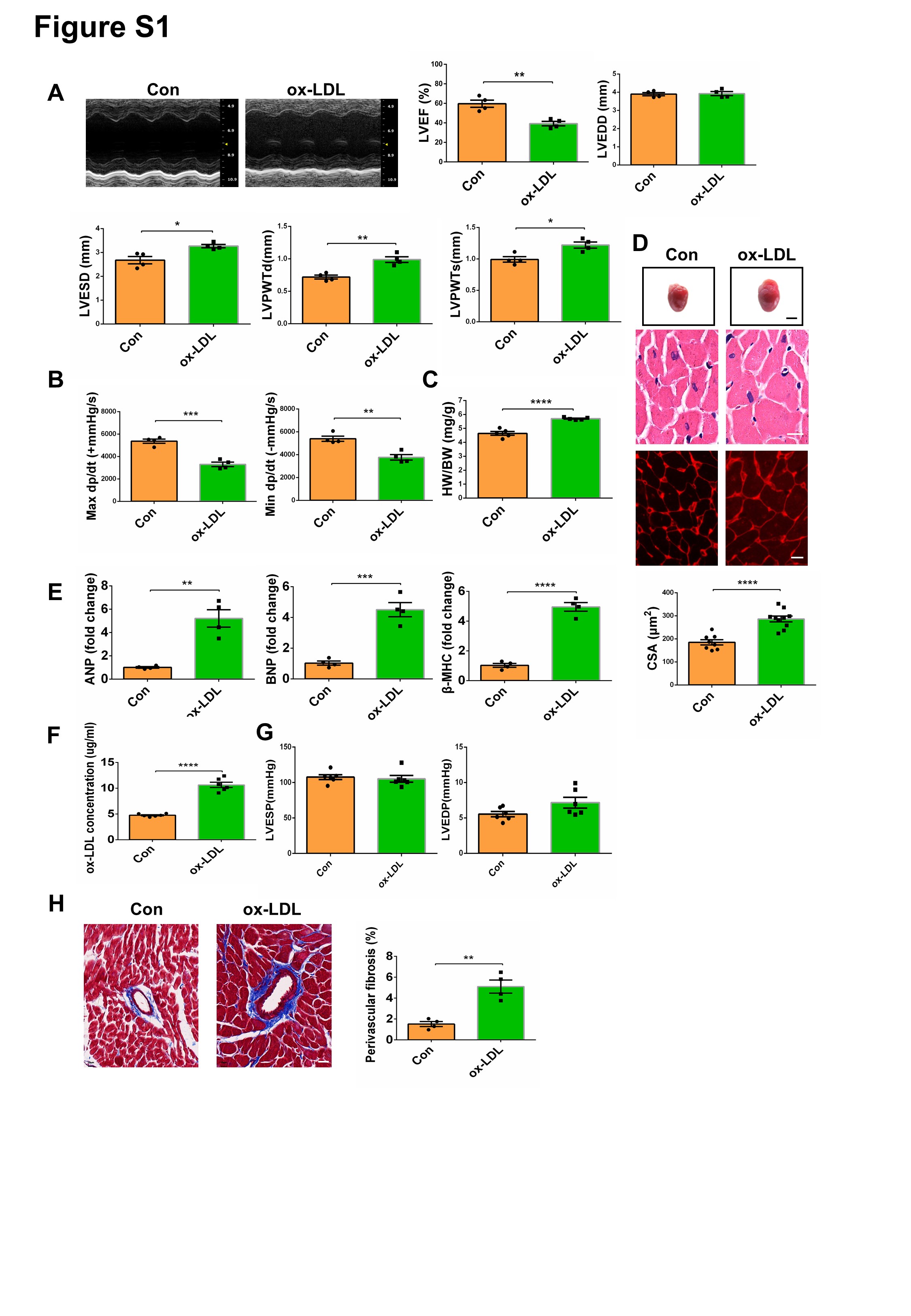

Supplement: Supplementary file 2 — Supporting information [file CTM2-12-e729-s007.JPG]

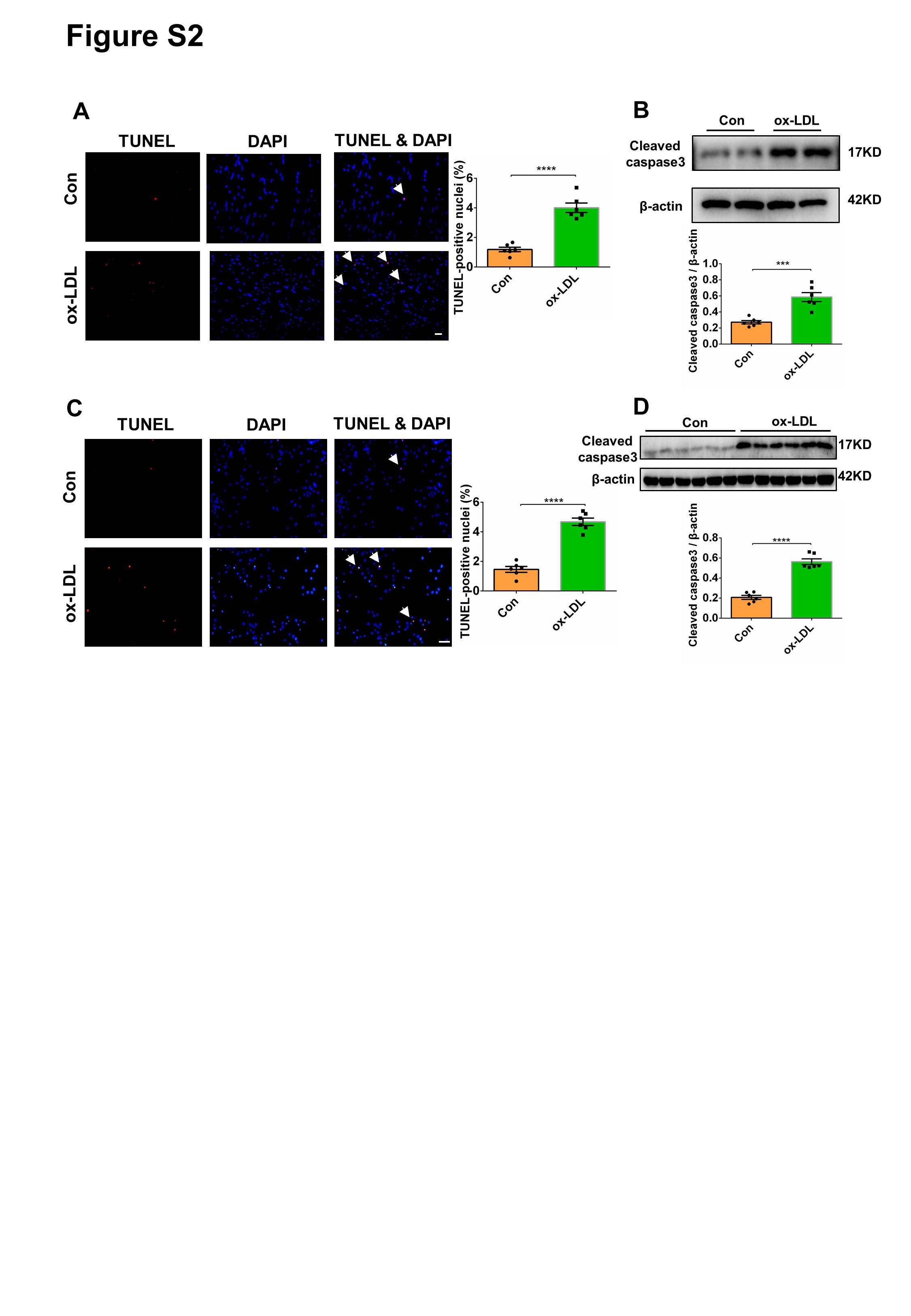

Supplement: Supplementary file 3 — Supporting information [file CTM2-12-e729-s005.JPG]

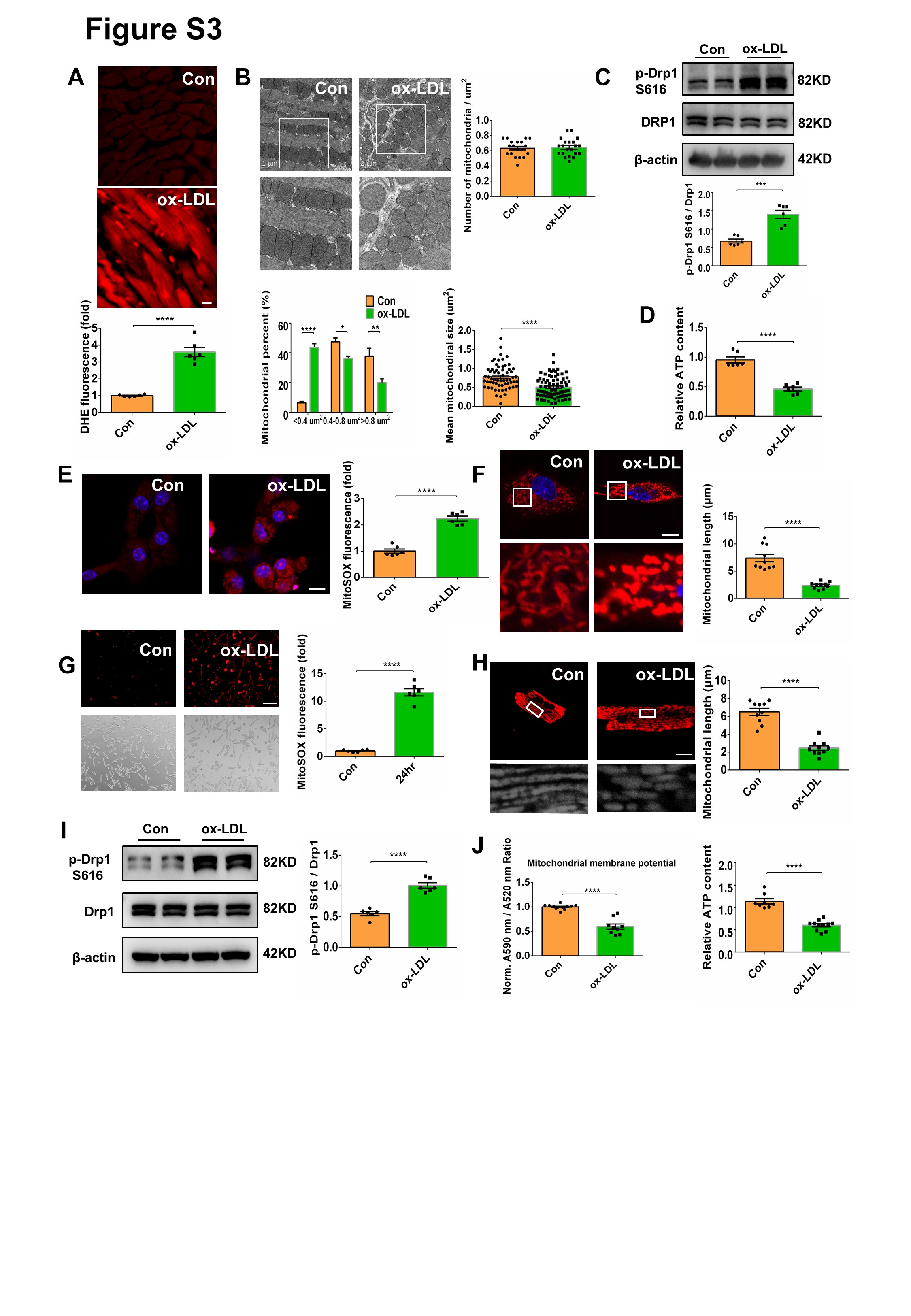

Supplement: Supplementary file 4 — Supporting information [file CTM2-12-e729-s001.JPG]

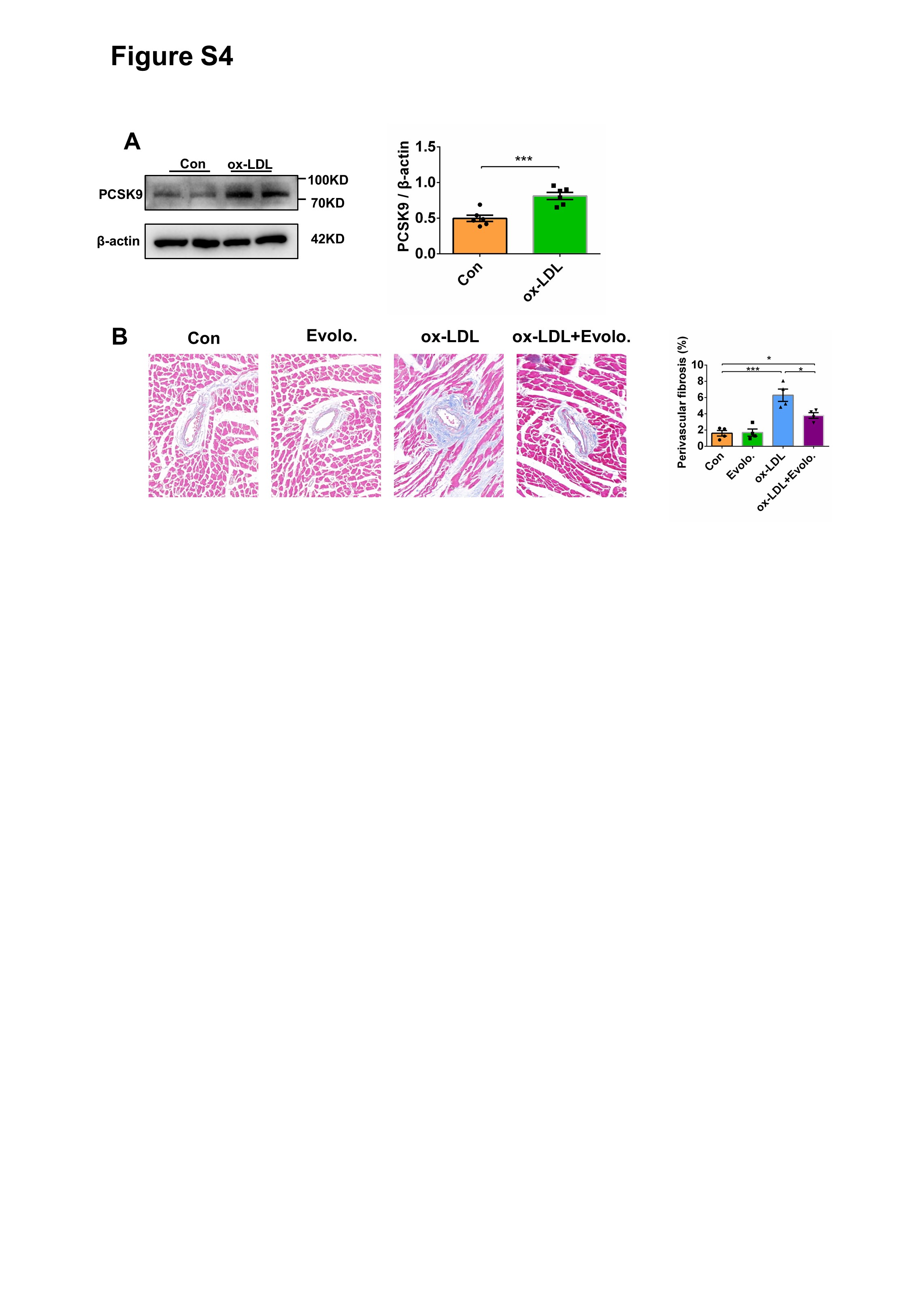

Supplement: Supplementary file 5 — Supporting information [file CTM2-12-e729-s002.JPG]

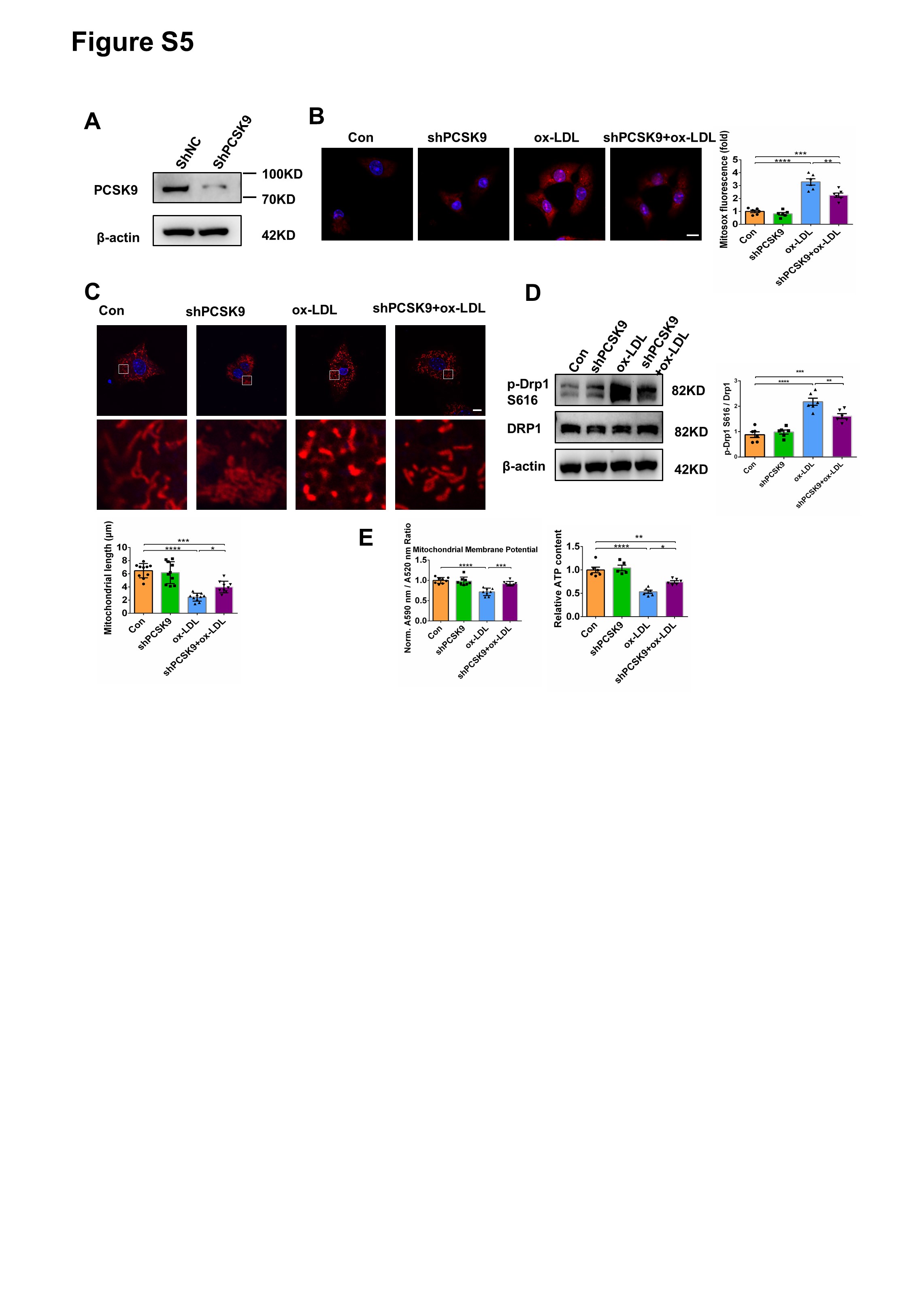

Supplement: Supplementary file 6 — Supporting information [file CTM2-12-e729-s006.JPG]

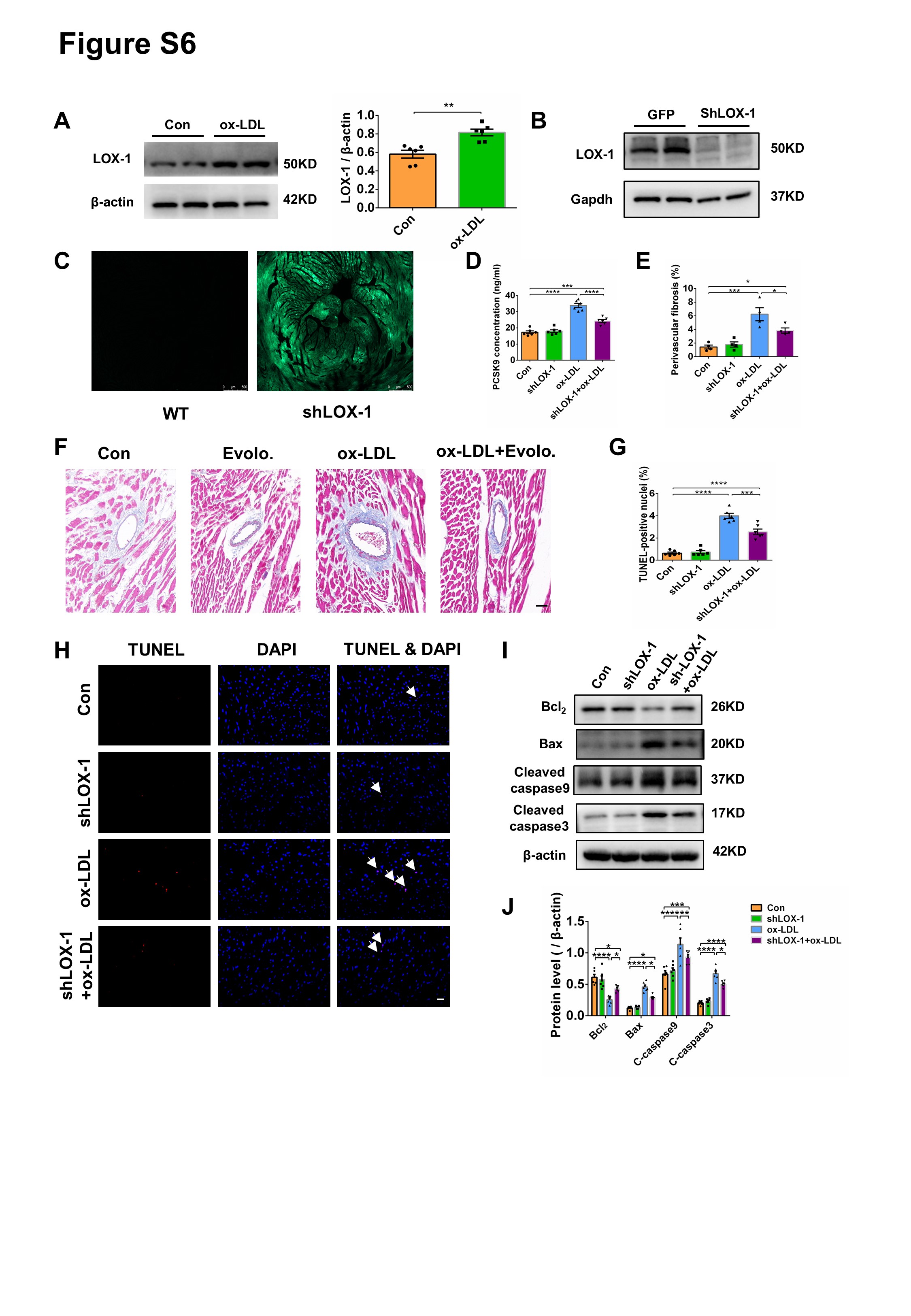

Supplement: Supplementary file 7 — Supporting information [file CTM2-12-e729-s008.JPG]

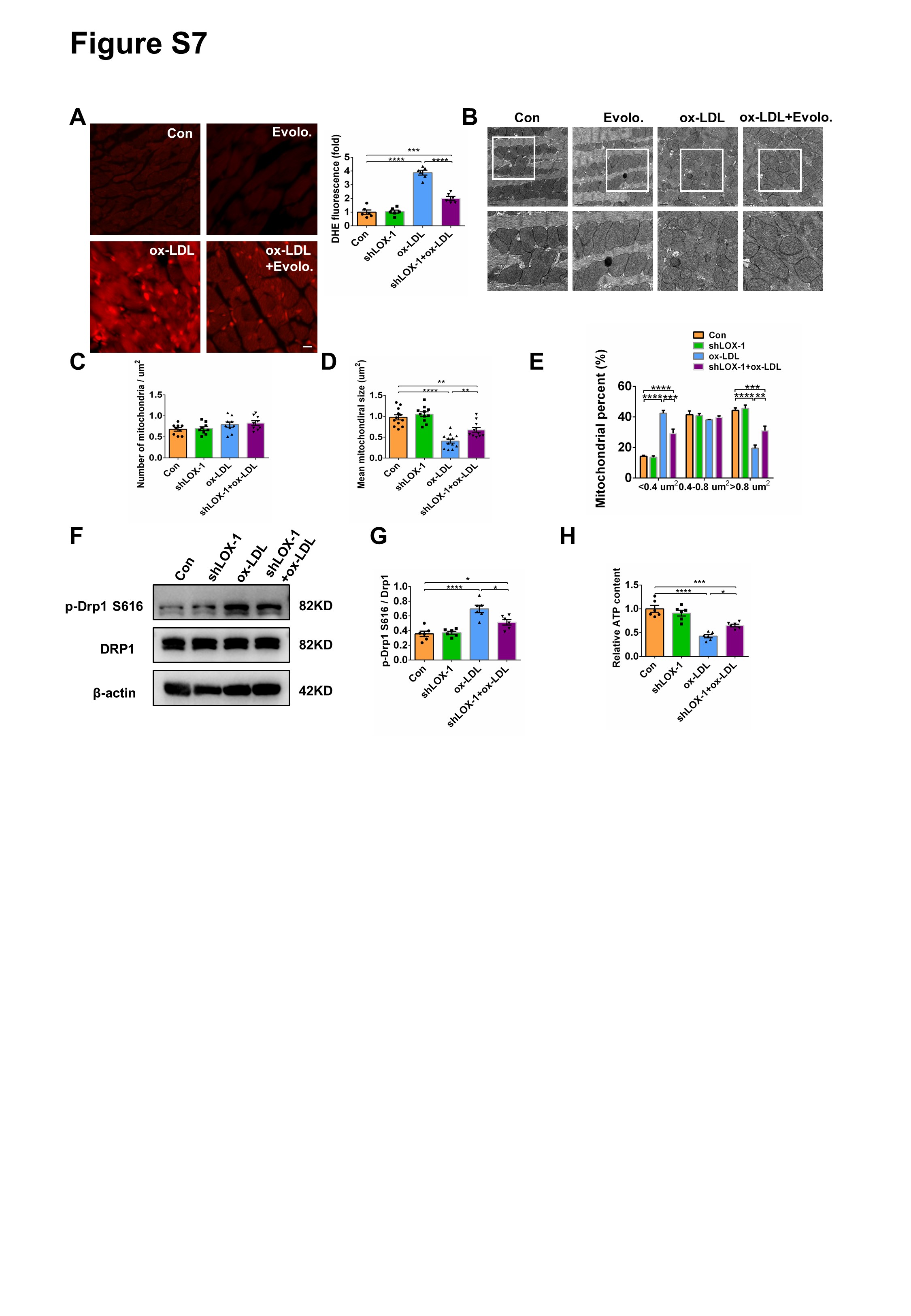

Supplement: Supplementary file 8 — Supporting information [file CTM2-12-e729-s003.JPG]
